# Italian pediatric experts' consensus statement on diagnosis and management of primary atopic disorders

**DOI:** 10.1111/pai.70225

**Published:** 2025-11-02

**Authors:** Fabio Cardinale, Ivan Taietti, Mayla Sgrulletti, Lucia Pacillo, Viviana Moschese, Caterina Cancrini, Raffaele Badolato, Michele Miraglia del Giudice, Gian Luigi Marseglia, Elena Chiappini, Riccardo Castagnoli, Primary Atopic Disorders Working Group, the Italian Society of Pediatric Allergy and Immunology (SIAIP), Salvatore Barberi, Anna Belloni Fortina, Roberto Berni Canani, Paolo Bottau, Carlo Caffarelli, Mauro Calvani, Antonella Cianferoni, Francesco Cinetto, Bianca Laura Cinicola, Emilia Cirillo, Francesca Conti, Laura Dotta, Marzia Duse, Silvia Federici, Alessandro Fiocchi, Elena Galli, Silvia Giliani, Giuliana Giardino, Cristiana Indolfi, Lucia Leonardi, Amelia Licari, Vassilios Lougaris, Sara Manti, Alberto Martelli, Domenico Minasi, Elio Novembre, Riccardo Papa, Claudio Pignata, Federica Pulvirenti, Maria Sangerardi, Annarosa Soresina, Mariangela Tosca, Alberto Giovanni Ugazio, Anna Maria Zicari

**Affiliations:** ^1^ Department of Pediatrics, Regional Referral Center in Pediatric Allergy, Immunology and Rheumatology, Giovanni XXIII Pediatric Hospital University of Bari Bari Italy; ^2^ Pediatric Unit, Department of Clinical, Surgical, Diagnostic, and Pediatric Sciences University of Pavia Pavia Italy; ^3^ Pediatric Clinic Fondazione IRCCS Policlinico San Matteo Pavia Italy; ^4^ Pediatric Immunopathology and Allergology Unit, Policlinico Tor Vergata University of Rome Tor Vergata Rome Italy; ^5^ Department of Systems Medicine University of Rome Tor Vergata Rome Italy; ^6^ Research Unit of Primary Immunodeficiencies, Unit of Clinical Immunology and Vaccinology Scientific Institute for Research and Healthcare (IRCCS) Bambino Gesù Children Hospital Rome Italy; ^7^ Molecular Medicine Institute “Angelo Nocivelli”, Department of Clinical and Experimental Sciences University of Brescia and ASST Spedali Civili Brescia Italy; ^8^ Department of Woman, Child and of General and Specialized Surgery University of Campania “Luigi Vanvitelli” Naples Italy; ^9^ Division of Pediatric Infectious Diseases Infective Diseases Anna Meyer Children's University Hospital Florence Italy; ^10^ Meyer Children's University Hospital, IRCCS Florence Italy

**Keywords:** early diagnosis, genetics, immune dysregulation, inborn errors of immunity, primary atopic disorders, severe atopic diseases, targeted treatment

## Abstract

**Background:**

Primary Atopic Disorders (PAD) represent a recently recognized subset of inborn errors of immunity (IEI), characterized by severe atopy driven by genetic mutations leading to dysregulated type 2 immune responses, excessive mast cell activation, and hyper production of IgE. In PAD patients, severe atopic manifestations, including eczema, asthma, food allergies, and eosinophilic gastrointestinal disorders, are often associated with other signs of immune dysfunction.

**Methods:**

Recognizing the need for standardized diagnostic and management guidelines for PAD, a Delphi‐based expert consensus was developed within the Immunology Committee of the Italian Society of Pediatric Allergy and Immunology (SIAIP). After a systematic review of the literature and the development of the clinical statements, 45 specialists from multiple pediatric subspecialties reached an agreement on key aspects of PAD classification, diagnosis, and treatment.

**Results:**

The consensus focuses on some red flags that could aid clinicians in suspecting PAD. The document also proposes a diagnostic work‐up to differentiate monogenic PAD from polygenic allergic conditions. It also emphasizes the importance of molecular pathway analysis to direct precision treatments, including biological drugs. Given the complexity of the field and the potential overlap between PAD and other IEI, the consensus recommends a multidisciplinary approach to diagnosis and treatment. The document establishes a framework for early recognition of PAD, integrating emerging genetic insights into clinical practice and promoting personalized therapeutic strategies.

**Conclusions:**

The present work is the first structured consensus to standardize PAD diagnosis and management among pediatric subspecialists, aiming to improve patient outcomes through early intervention and tailored therapies.

**Key message**

Primary Atopic Disorders (PAD) are a recently recognized group of inborn errors of immunity (IEI) characterized by severe allergic manifestations, frequently associated with immune dysregulation and hyper IgE/severe eosinophilia, often presenting early in life but potentially extending into adulthood. Due to their heterogeneous and overlapping features with common atopic diseases, PAD diagnosis is often delayed. This consensus provides a standardized work‐up and a core set of clinical and laboratory red flags to differentiate PAD from polygenic multifactorial allergic conditions. Understanding the molecular mechanisms underlying PAD is crucial. This document highlights how genetic testing and pathway analysis guide targeted therapeutic interventions.

## 1 INTRODUCTION

Inborn errors of immunity (IEI) are a heterogeneous expanding group of monogenic disorders with a broad spectrum of clinical manifestations ranging from recurrent infections to lymphoproliferation and malignancy.^1^ Increasing evidence suggests that immune dysregulation, including severe atopy, may be a distinctive clinical feature associated with several IEI.^2^, ^3^, ^4^ In 2018, Lyons and Milner coined the term Primary Atopic Disorder (PAD) to define heritable genetic traits, presenting with dysregulated type 2 allergic effector responses, in some cases independent of IgE sensitization. These genetic disorders encompass a wide range of clinical manifestations as a consequence of abnormal mast cell activation, chronic T‐helper type 2 (Th2) and eosinophil‐mediated allergic inflammation, and exuberant immunoglobulin E (IgE) production.^5^

Common atopic clinical features of PAD include eczema, allergic rhinitis, asthma, eosinophilic gastrointestinal disorders (EGID), and food allergy (FA), frequently associated with very high levels of IgE and eosinophils in blood and tissue hypereosinophilia. Different and more complex atopic phenotypes are continuously being described,^6^, ^7^ and some authors estimate that PAD covers more than 48 monogenic disorders.^8^ The main pathogenic pathways involve cytokine signaling, T cell receptor (TCR) signaling, actin cytoskeleton breakdown, tolerance failure, intrinsic mast cell functions, and skin barrier perturbation.^9^ Nowadays, elucidating the pathogenetic mechanisms of severe monogenic allergy is crucial for guiding a precision medicine approach and providing insights into treating more common allergic conditions of potential multifactorial origin.

Recently, to help clinicians in distinguishing monogenic PAD from polygenic disorders, Castagnoli et al.^10^ grouped PAD according to the dominating pattern of clinical symptoms as follows: (1) hyper‐IgE syndromes (HIES); (2) Omenn syndrome (OS); (3) Wiskott‐Aldrich syndrome (WAS) and WAS‐like conditions; (4) immune dysregulation, polyendocrinopathy, enteropathy, X‐linked (IPEX) and IPEX‐like conditions; (5) Caspase recruitment domain (CARD) proteins – B‐cell CLL/lymphoma 10 (BCL10) – MALT1 paracaspase (MALT1) (CBM) – opathies; (6) miscellaneous IEI with predominant allergic manifestations. Moreover, a distinct subgroup of PAD is represented by mast cell hyperreactivity syndromes, prototypically represented by mastocytosis, due to somatic or germline gain‐of‐function *KIT* mutations.

However, it is now clear that overlapping clinical features are common among PAD, making it difficult for clinicians to distinguish among different entities relying only on clinical grounds. Also, common atopic disorders may share variant alleles with less penetrance in the same genes causing PAD.^11^, ^12^, ^13^, ^14^ Furthermore, environmental factors, including diet, infections, and the commensal microbiota, have been hypothesized as possible exogenous factors conditioning clinical phenotype and possibly the age of presentation of PAD.^12^

There is a compelling need among pediatric subspecialty clinicians with different perspectives on the same disease to harmonize as much as possible the correct management of these rare diseases, frequently presenting with common but difficult‐to‐treat type 2 symptoms. In situations where no gold standard or formal consensus exists, peer opinions can serve as a recognized approach to advance scientific knowledge based on clinical experience and authoritative viewpoints and support clinical decisions.^15^

Pursuing this goal, the Immunology Committee of the Italian Society of Pediatric Allergy and Immunology (SIAIP) aimed to develop an evidence‐based expert opinion consensus statement using a Delphi approach to manage patients with PAD. This consensus was developed by 45 experts in the field of IEI and included pediatric immunologists, allergologists, pulmonologists, dermatologists, gastroenterologists, and geneticists involved in the management of PAD at tertiary referral centers across Italy.

The Delphi method is a recognized formal technique to build consensus in various fields. This process entails individual expert consultation through questionnaires and collecting anonymous feedback through interaction.^15^, ^16^ The Delphi is an iterative multistage process that combines opinions into group consensus. The Delphi technique facilitates consensus building and promotes a process of cognitive reflection. Participants can revise their judgments based on emerging insights or previously overlooked information through iterative rounds and structured feedback. Therefore, the Delphi method allows participants to reconsider their assessments considering new information.^16^ This process is particularly advantageous in addressing complex topics, such as PAD, where multidisciplinary knowledge and evolving perspectives must be integrated to refine diagnostic criteria and management approaches.^17^

## 2 MATERIALS AND METHODS

We adhered to the ACCORD (ACcurate COnsensus Reporting Document) guidelines for reporting the methodology and results of the present consensus.^18^

The work, which was conducted from January 2024 to December 2024, involved 45 Italian experts with relevant clinical experience in managing patients with PAD operating in pediatric hospitals and outpatient clinics.

### 2.1 Exploratory Phase

In 2024, the SIAIP Immunology Committee identified the need to integrate current evidence on managing PAD among pediatric subspecialties. The Italian Pediatric Expert Task Force on Primary Atopic Disorders (F.C., I.T., M.S., L.P., V.M., C.C., R.B., R.C.) was nominated. This Task Force identified a group of physicians involved in PAD care who constituted the panelist board. Experts were pediatric immunologists, allergologists, gastroenterologists, pulmonologists, dermatologists, and geneticists, working in academic institutions or hospital settings with at least 5 years of clinical experience.

The following criteria were chosen for expert panel selection: clinical experience in the field of PAD; working in hospitals and academic institutions throughout the Italian territory; playing an active role in national or international task forces as a part of qualified pediatric scientific societies [Italian Society of Pediatric Allergy and Immunology (SIAIP), Italian Primary Immune Deficiency Network (IPINet), Italian Society of Infantile Respiratory Diseases (SIMRI), Italian Society of Pediatric Dermatology (SIDERP), Italian Society of Pediatric Gastroenterology, Hepatology and Nutrition (SIGENP), Italian Society of Pediatrics (SIP), Italian Society for Pediatric Research (SIRP)].

Before starting the consensus exercise, all panelists were asked to disclose any conflicts of interest. The coordinator (F.C.), reworkers (I.T., R.C.), and research methodology consultant (E.C.) determined that the lack of conflicts of interest among all the panelists adequately mitigated the potential risk of bias.

**FIGURE 1:**
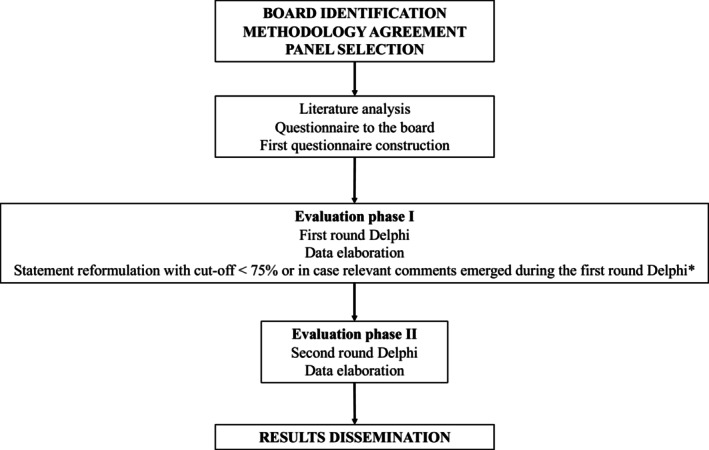
Delphi method flow chart. *These relevant comments were discussed during a face‐to‐face meeting among the advisory board and the statements were reformulated according to the agreement in this meeting.

The Delphi methodology was used to reach a consensus (Figure 1). Therefore, a 4‐tier approach was adopted: (1) literature searching, selection of relevant articles, and sharing among panelists belonging to the advisory board; (2) identifying areas of intervention from the advisory board and developing a core of statements; (3) evaluation and voting for statements among all panelist members (2 rounds of voting); (4) final approval of the document from all panelists.

### 2.2 Analytical Phase

A literature search was performed in May 2024 on:

1. PubMed database using the following keywords:
  - ((Inborn errors of immunity) OR (primary immodef*)) AND ((atopy OR atopic dermatitis) OR (atopy OR food allergy) OR (atopy OR asthma) OR (atopy OR eosinophilic gastrointestinal disorders)).
  - ((((((((atopic dermatitis[MeSH Terms]) OR (atopic eczema[MeSH Terms])) OR (severe eczema[MeSH Terms])) OR (asthma[MeSH Terms])) OR (severe asthma[MeSH Terms])) OR (food allergy[MeSH Terms])) OR (eosinophilic gastrointestinal disease[MeSH Terms])) OR (eosinophilic esophagitis[MeSH Terms])) OR (atopic phenotypes[MeSH Terms])) AND (((((((((((((((((((((inborn errors of immunity[MeSH Terms]) OR (IEI[MeSH Terms])) OR (primary immunodeficiencies[MeSH Terms])) OR (PID[MeSH Terms])) OR (primary atopic disorders[MeSH Terms])) OR (Hyper‐IgE syndromes[MeSH Terms])) OR (HIES[MeSH Terms])) OR (STAT3 deficiency[MeSH Terms])) OR (Job syndrome[MeSH Terms])) OR (DOCK8 deficiency[MeSH Terms])) OR (Comel‐Netherton syndrome[MeSH Terms])) OR (Omenn syndrome[MeSH Terms])) OR (Wiskott‐Aldrich syndrome[MeSH Terms]))) OR (Immunodysregulation, polyendocrinopathy, enteropathy, X‐linked[MeSH Terms])) OR (IPEX[MeSH Terms])) OR (CBM‐opathies[MeSH Terms])) OR (NEMO deficiency[MeSH Terms])) OR (JAK1 gain of function[MeSH Terms])) OR (STAT5b gain of function[MeSH Terms])) OR (STAT6 gain of function[MeSH Terms])).
2. Embase database using the following keywords:
  - (“inborn errors of immunity”/exp. OR “inborn errors of immunity” OR (inborn AND errors AND of AND (“immunity”/exp. OR immunity)) OR (primary AND immunodef*)) AND (“atopic dermatitis”/exp. OR “atopic dermatitis” OR (atopic AND (“dermatitis”/exp. OR dermatitis)) OR “food allergy”/exp. OR “food allergy” OR ((“food”/exp. OR food) AND (“allergy”/exp. OR allergy)) OR “asthma”/exp. OR asthma OR “atopy”/exp. OR atopy OR “eosinophilic gastrointestinal disorders”/exp. OR “eosinophilic gastrointestinal disorders” OR (eosinophilic AND gastrointestinal AND (“disorders”/exp. OR disorders)))

**FIGURE 2:**
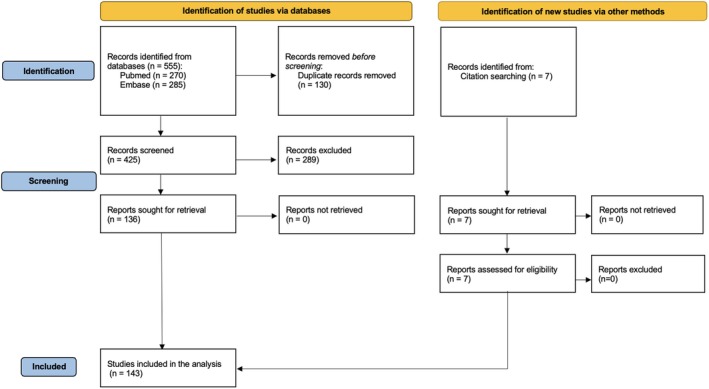
Literature search strategy flow diagram.

The search included observational studies (prospective or retrospective cohorts, case–control or cross‐sectional studies), systematic reviews and meta‐analyses, case reports/series, and guidelines published between 2014 and 2024. Publications in languages other than English, letters, and editorials were not included. One hundred thirty‐six articles were considered relevant for analysis (Figure 2). Furthermore, seven additional studies were identified through “citation searching” in the relevant sources.

### 2.3 Delphi approach^16^

Based on a literature search, a questionnaire was prepared by the Italian Pediatric Expert Task Force on Primary Atopic Disorders (F.C., I.T., M.S., L.P., V.M., C.C., R.B., R.C.) who identified 11 areas of interest and elaborated 24 statements (Table 1). The questionnaires were administered to the experts via a web platform that guaranteed anonymity and the exclusion for the data manager to associate the single questionnaire with the compiler. Literature supporting the questionnaire was shared when needed and made available to all panelists through common free‐access web‐based box tools.

**TABLE 1:** Summary of the results of the two Delphi consultations (first and second round).

First round statement	Mean	Agreement %	Second round statement	Mean	Agreement %
General definition					
PAD refer to a group of rare human monogenic IEI with severe allergic or atopic effector–related symptoms as a substantial feature. Abnormal allergic immune responses could be directed to various environmental triggers, regardless of sensitization leading to complex and heterogeneous phenotypes. Infectious and non‐infectious immunodeficiency features (e.g, recurrent, severe, and/or atypical infections, autoimmunity, inflammation, lymphoproliferation, malignancies) may be variably associated.	4.7	100			
Pathogenesis					
2 PAD encompass a group of genetic disorders resulting in pathologic mast cell activation and degranulation, persistent allergic inflammation mediated by T‐helper (Th) 2 cells and eosinophils, as well as exaggerated immunoglobulin (Ig) E production.	4.5	100			
3 Knowledge of the affected pathway may be useful to identify potential target therapies.	4.9	100			
Classification					
4 PAD may be classified, according to pathogenetic mechanisms in: Skin barrier dysfunction; Altered cytokine signaling; Impaired T cell receptor signaling and/or cytoskeletal remodeling; Metabolic disturbance; Mast cell dysregulation; T cell repertoire restriction; Tolerance failure.	4.5	100			
5 PAD can be categorized as a subgroup of Atopy‐Associated Inborn Errors of Immunity (AA‐IEI).	4.4	97.6	5 In future classifications, Inborn Errors of Immunity with atopic phenotypes could be categorized as a distinct subgroup among Inborn Errors of Immunity with immune dysregulation (Table 4 of IUIS Classification).	90.5	4.3
General clinical features					
6 Although patients suffering from PAD typically manifest early‐onset atopic disease, usually at birth or in the first months of life (including Omenn Syndrome, OS), and in general during the pediatric age, PAD may also manifest until adulthood and coexist within families, with wide phenotypic variability.	4.6	97.6			
7 Atopic manifestations of PAD are often severe and usually not responsive to standard therapy (e.g., severe and recalcitrant eczema, severe asthma, multiple food allergies, anaphylaxis, unresponsive eosinophilic gastrointestinal disease), with frequent need for targeted therapies. Several associated clinical features (congenital ichthyosis, skeletal abnormalities, neurodevelopmental delay, diarrhea, endocrinopathy, bleeding, and/or failure to thrive) strengthen the suspicion of PAD. Recurrent/severe infections (especially due to opportunistic pathogens and Herpesviridae, including CMV, EBV, and HHV‐6) are frequent, but may also be absent.	4.7	100			
8 OS is one of the most severe and life‐threatening manifestations of PAD that has to be ruled out before starting the diagnostic work‐up. OS is defined as generalized erythroderma (skin inflammation affecting more than 90% of the body surface), lymphadenopathy, hepatosplenomegaly, profound eosinophilia, chronic persistent diarrhea, failure to thrive, and combined immune‐deficiency (CID).	4.3	85.7	8 OS is a severe and life‐threatening manifestation of PAD, typical of SCID, that should be quickly ruled out in a newborn or infant with suspected PAD. OS is defined as generalized erythroderma with skin inflammation affecting more than 90% of the body surface, lymphadenopathy, hepatosplenomegaly, profound eosinophilia, chronic persistent diarrhea, failure to thrive, and CID.	4.4	95.2
9 The presence of other affected family members, including a family history of IEI and/or familial severe atopic diathesis, and/or a family history of consanguinity could raise the index of suspicion for PAD. Inheritance patterns may differ among distinct IEIs with atopic phenotypes.	4.6	100			
Laboratory evaluation					
10 High levels of Th2 biomarkers and associated immunological abnormalities are of extreme importance in the diagnostic work‐up of IEI with severe atopic features to highlight some indicative features of PAD. The following exams are mandatory in ruling out PAD in children: Complete blood cell count with differential (CBC); Serum immunoglobulin (Ig) [IgA, IgM, IgG, IgE], and IgG subclasses dosage; Extended T and B cell immunophenotype (CD3, CD4, CD8, CD19, CD56, Treg, Th17, T follicular cells, HLA‐DR expression); Specific antibody levels against recall antigens (vaccines, ABO group, others); Allergy testing (according to patient's medical history): skin prick test and/or allergen‐specific IgE measurement; Markers of organ‐specific and tissue‐specific autoimmunity (according to patient's medical history).	4.5	92.8			
11 In the context of severe, especially multiorgan, allergic disease the following laboratory abnormalities should be considered for the suspicion of PAD: cytopenias (neutropenia/thrombocytopenia/anemia); lymphopenia; increased absolute number of eosinophilic granulocytes (severe if >5000 cells/mm^3^); decreased mean platelet volume (MPV); very high levels of serum IgE (>1000 kU/L, in the first 3 months of life; >2000 kU/L in children 3–12 months; >5000 kU/L after 12 months of age); one or more immunoglobulin isotype deficiency (any combination); low specific antibody levels against protein and polysaccharide antigens (especially after booster vaccination if vaccine antibody response is considered); low Th17 cell counts; low CD3+CD25+ regulatory T cell counts; very high level of sensitization against multiple environmental allergens (often combined); high levels of autoimmunity markers in blood (often precocious and atypical); high levels of tryptase in blood.	4.3	88.1			
PAD and skin					
12 Atopic dermatitis (AD) represents the typical skin findings of severe atopic disorders as well as of PAD. However, other skin features can be present (ichthyosis erythroderma, *trichorrhexis invaginata*, ectodermal dysplasia among others).	4.5	100			
13 The following peculiar skin findings may help in differentiating IEI with atopic phenotypes from severe AD: Congenital/early onset (even neonatal) erythroderma or neonatal eczematous rash. Congenital ichthyosis (early‐onset generalized rash that evolves into severe ichthyosis). Bamboo hair (*trichorrhexis invaginata*). Atypical localization, extension, and features of lesions (e.g., thickened texture of the facial skin, retro auricular fissures, and severe folliculitis of the axillae and groin). Chronic mucocutaneous candidiasis (CMC). Cold skin abscesses. Warts. Ectodermal dysplasia. Severe urticarial rash, chronic urticaria, and refractory cold urticaria, with no itching but with pins‐and‐needles‐like pain in affected skin areas Striate palmoplantar keratoderma	4.6	97.6			
PAD and respiratory system					
14 PAD should be suspected in severe asthma (also in case of low Th2 endotype), with frequent exacerbations, treatment unresponsiveness and/or chronic lung disease (including the presence of bronchiectasis). In particular, its occurrence in association with other atypical atopic diseases and/or other features of immune dysregulation should raise the suspicion of PAD and should prompt an immunologist consultation.	4.5	97.6			
PAD and food allergies (FA)					
15 Multiple and/or severe FA (e.g., anaphylaxis) and/or food protein‐induced enterocolitis syndrome (FPIES), associated with other atopic manifestations, even severe, and/or other features of immune dysregulation should raise the suspicion of PAD and should prompt an immunologist consultation.	4.3	92.8	15 Multiple and/or severe FA, (e.g., anaphylaxis) including food protein‐induced enterocolitis syndrome (FPIES), when associated with other atopic manifestations, especially if severe, and/or other features of immune dysregulation and/or increased susceptibility to infections should raise the suspicion of PAD and should prompt an immunologist consultation.	90.5	4.3
PAD and eosinophilic gastrointestinal disorders (EGID)					
16 EGID associated with severe eosinophilia (>5000 cells/mm^3^) and multiorgan involvement with/without atopic diathesis should be considered as a potential manifestation of PAD. EGIDs are clinically heterogeneous diseases that are more commonly found in patients with different IEI.	4.4	97.6			
Warning signs					
17 Warning signs for PAD include: Severe atopic manifestations (especially when multiple) with very early onset (at birth or in the first months of life) and unresponsiveness to standard treatment. Recurrent and severe infections due to atypical pathogens (e.g., herpes viruses, HPV, fungi). Short stature and/or failure to thrive. Lymphoproliferation and/or malignancies. Poli‐autoimmunity. Severe diarrhea (even with eosinophilic infiltration) and or enteropathy. Poli‐endocrinopathy (with particular regard to early‐onset, eventually neonatal, T1DM). Vascular and connective tissue abnormalities. Positive family history for IEIs/consanguinity. Hematological conditions (e.g., purpura, cytopenia). Very high levels of total IgE (>1000 kU/L, in the first 3 months of life; >2000 kU/L in children 3–12 months; >5000 kU/L after 12 months of age) Increased absolute number of eosinophils in blood (>5000 cells/mm^3^).	4.4	90.5			
18 The combination of severe atopic manifestations (especially when multiple) with two or more of the following clinical and laboratory findings increases the possibility of a PAD: Very early onset (<3 months). Multiple atopic diseases. Recurrent and severe infections due to atypical pathogens (e.g., herpes viruses, HPV, fungi). Short stature and / or failure to thrive. Lymphoproliferation and/or malignancies. Poli‐autoimmunity. Severe persistent diarrhea (particularly when associated with gut eosinophilic infiltration) and/or enteropathy. Poli‐endocrinopathy (with particular regard to early‐onset [possibly neonatal], T1D). Vascular and connective tissue abnormalities. Positive family history for IEIs/consanguinity. Very high levels of total IgE (>1000 kU/L, in the first 3 months of life; >2000 kU/L in children 3–12 months; >5000 kU/L after 12 months of age). Increased absolute number of eosinophils in blood (>5000 cells/mm^3^). Cytopenia along one or more lineages.	4.5	95.2			
19 The combination of severe atopic manifestations (especially when multiple), including urticaria, angioedema and anaphylaxis, with one or more of the following laboratory findings increases the possibility of a PAD: Very high levels of total IgE (any age) >10,000 kU/L. Increased absolute number of eosinophils in blood (any age) >10,000 cells/mm^3^. Basal high levels of tryptase and/or histamine in blood.	4.2	80.9	19 The combination of severe atopic manifestations (especially when multiple), including urticaria, angioedema and anaphylaxis, with one or more of the following laboratory findings, once a malignant hematological disorder has been excluded, increases the possibility of a PAD: Very high levels of total IgE (any age) >10,000 kU/L increased absolute number of eosinophils in blood (any age) >10,000 cells/mm^3^ basal high levels of tryptase and /or histamine in blood. However, lower values of these biomarkers may be commonly found in IEI associated with atopy and do not rule out a PAD.	4.4	92.9
20 Normal levels of total IgE do not rule out a diagnosis of PAD.	4.4	100			
Genetics					
21 Identification of warning signs for PAD should prompt an immunologic consultation, prior to genetic testing. Moreover, the indication and type of genetic testing should be given as a joint geneticist and clinical immunologist evaluation [expert opinion].	4.6	95.2			
22 Good clinical response to specific target immunomodulatory therapies may support the hypothesis of a monogenic rather than multifactorial nature of the defect.	3.9	78.6	22 Clinical response to specific target disease modifying therapies is expected once genetic diagnosis has been established and pathogenetic mechanisms elucidated. [expert opinion].	4.2	88.1
23 As for other IEI, novel disease‐causing genes are continuously identified for PAD. For these reasons, an initial negative result does not rule out PAD diagnosis and patients should be periodically re‐evaluated according to the most recent knowledge.	4.7	100			
24 Functional tests should be used to confirm PAD diagnosis and evaluate the pathogenic role of variants of uncertain significance (VUS) eventually detected. Functional tests could also drive a therapeutic approach while waiting for the genetic analysis results.	4.5	95.2			

Panelists were asked to score each statement on the following scale: 1, strong disagreement; 2, fair disagreement; 3, no opinion; 4, fair agreement; 5, strong agreement. For the analysis of the results, responses were categorized as negative (score 1–2), neutral (score 3), and positive (score 4–5). For each statement, the average score and the percentage of voters who gave positive responses were considered, and the cut‐off level for consensus was set at 85% agreement (considering neutral and positive scores: 3–4–5). After the first round, the board evaluated the responses to identify areas of divergence and acquire further information to improve the survey. Modified statements were then developed and submitted until a satisfactory agreement was achieved.

## 3 RESULTS

A panel of 45 experts distributed all over the country was asked to fill out the questionnaire (Table 1). The results of the first and second rounds are presented in Table 1. After the first round, a high level of consensus for all the statements, with average scores ranging from 3.9 to 4.9 and agreement ranging from 78% to 100%, was observed. The five questions that did not reach the predetermined consensus level were modified and discussed again in the second round.

### 3.1 Definition of Primary Atopic Disorders (PAD)

**Statement #1**. PAD refer to a group of rare human monogenic IEI with severe allergic or atopic effector–related symptoms as a substantial feature. Abnormal allergic immune responses could be directed to various environmental triggers, regardless of sensitization, leading to complex and heterogeneous phenotypes. Infectious and non‐infectious immunodeficiency features (e.g, recurrent, severe and/or atypical infections, autoimmunity, inflammation, lymphoproliferation, malignancies) may be variably associated.

*Comment*: Increasing evidence suggests that allergic immune dysregulation may be more widespread in IEIs than previously reported.^19^ The study of monogenic causes of IEIs has led to significant advances in our understanding of immune system function, unveiling that some IEIs have comorbid phenotypes associated with type 2 or allergic effector responses as part of the clinical spectrum of disease. In these monogenic disorders, allergic phenotypes can occur independently, and they may not even present with overt immunodeficiency, increasing the heterogeneity of the clinical spectrum.^9^, ^10^, ^20^, ^21^, ^22^, ^23^

### 3.2 Pathophysiology

**Statement #2**. PAD encompass a group of genetic disorders resulting in pathologic mast cell activation and degranulation, persistent allergic inflammation mediated by T‐helper (Th) 2 cells and eosinophils, as well as exaggerated immunoglobulin (Ig) E production.

*Comment*: Several genes involved in IEIs (including many disease‐causing genes resulting in combined immune deficiencies (CIDs), defects in intrinsic and innate immunity, and diseases of immune dysregulation^24^) have been identified as related to the development of atopy. Moreover, several underlying identified pathways specifically promoting allergic phenotypes in PAD have been identified involving adaptive immune cells (i.e., impaired TCR signaling and cytoskeletal remodeling, T cell repertoire restriction, altered cytokine signaling, tolerance failure, and metabolic disturbance), mast cells (i.e., altered mast cell activation), and epidermal keratinocytes (i.e., skin barrier dysfunction).^5^, ^9^, ^25^, ^26^ When performed, comprehensive cytokine profiling in patients with PAD reveals unique and distinctive expression patterns of various inflammatory cytokines when compared to patients with AD. This indicates disease‐specific disruptions in multiple cellular processes and pathways, which lead to increased susceptibility to infections, immune tolerance breakdown, and allergies.^27^

**Statement #3**. Knowledge of the affected pathway may be useful to identify potential target therapies.

*Comment*: Genetic characterization or identifying the specific altered molecular pathway may be helpful for diagnostic, therapeutic, and prognostic purposes.^25^ Although for some PAD, hematopoietic stem cell transplantation (HSCT) or gene therapy still represents the only curative drug option, others can be appropriately managed with novel or repurposed therapeutics that precisely target the defective molecular pathways, improving patient outcomes.^12^, ^28^, ^29^, ^30^, ^31^ Understanding the molecular basis of these diseases allows the development of targeted therapies that can reverse atopic inflammation and restore immune balance in affected patients.^13^, ^32^, ^33^, ^34^, ^35^, ^36^

### 3.3 Classification

**Statement #4**. PAD may be classified, according to pathogenetic mechanisms in:

- Skin barrier dysfunction;
- Altered cytokine signaling;
- Impaired T cell receptor signaling and/or cytoskeletal remodeling;
- Metabolic disturbance;
- Mast cell dysregulation;
- T cell repertoire restriction;
- Tolerance failure.

*Comment*: Due to their heterogeneity, a clinically based classification has been proposed grouping PAD into six clinical phenotypes. In addition, many experts agree that PAD could also be classified according to the underlying pathogenic mechanism with significant implications for targeted therapies.^5^, ^9^, ^25^, ^37^

**Statement #5**. In future classifications, Inborn Errors of Immunity with atopic phenotypes could be categorized as a distinct subgroup among Inborn Errors of Immunity with immune dysregulation [Table 4 of International Union of Immunological Societies (IUIS) Classification].

*Comment*: Due to their predominant manifestations of severe difficult‐to‐treat type 2 symptoms, it is conceivable that PAD in the near future could be included as distinct entities among “Diseases of Immune Dysregulation” (Table 4) of the IUIS classification.^1^, ^38^, ^39^, ^40^ The rationale for this new categorization is the perspective that new disorders are identified among this expanding group of IEIs, most of which are presently included as “hyper‐IgE syndromes” or “other defects” in Table 2 in the IUIS classification. Further, some new (e.g., *STAT6* GOF mutations) and old (e.g., *CDSN* and *DSG1* gene mutations) clinical entities, which collectively can be included among Lyons and Milner's definition of PAD, are not included in the IUIS classification. Another reason is that some PAD (e.g., *STAT5b* GOF, *KIT*, and *ADGRE2* gene mutations) could have a prominent clinical and laboratory feature of hypereosinophilia or aberrant mast cell activation, which do not fit with the definition of “hyper‐IgE syndromes” in the IUIS classification. However, the panelists agree that the attempt to group within a unique clinical entity may not be representative of all phenotypes of PAD and that overlapping features exist with other categories of IEI.

### 3.4 General clinical features

**Statement #6**. Although patients suffering from PAD typically manifest early‐onset atopic disease, usually at birth or in the first months of life (including Omenn Syndrome, OS), and in general during the pediatric age, PAD may also manifest until adulthood and coexist within families, with wide phenotypic variability.

*Comment*: Most PAD occur early, typically at birth or within the first few months of life, often with severe and multiple atopic diseases, in most cases associated with other clinical signs of immune dysfunction, such as abnormal susceptibility to infections or autoimmunity.^41^ Severe combined immunodeficiencies (SCID), leaky SCID, and OS were among the first known IEIs presenting with early‐onset atopic/Th2‐mediated manifestations.^25^ Many individuals may receive a diagnosis during adulthood due to the progression of symptoms or the late onset of their condition. Therefore, clinicians should consider PAD in both adult and pediatric patients. It is important to note that PAD is not limited to infants, as adults do not represent a negligible part of patients.^12^, ^42^, ^43^, ^44^, ^45^ Plausible explanations include gene‐specific mutations and gene–environment (e.g., microbiome, infection, diet, and stress) interactions that likely contribute to variable penetrance and expressivity (type, severity, and age of onset of symptoms) of the disease.^46^

**Statement #7**. Atopic manifestations of PAD are often severe and usually not responsive to standard therapy (e.g., severe and recalcitrant eczema, severe asthma, multiple food allergies, anaphylaxis, unresponsive eosinophilic gastrointestinal disease), with a frequent need for targeted therapies. Several associated clinical features (congenital ichthyosis, skeletal abnormalities, neurodevelopmental delay, diarrhea, endocrinopathy, bleeding, and/or failure to thrive) strengthen the suspicion of PAD. Recurrent/severe infections (especially due to opportunistic pathogens and *Herpesviridae*, including CMV, EBV, and HHV‐6) are frequent, but may also be absent.

*Comment*: In some IEIs, allergic symptoms can be the most prominent clinical features. These symptoms may include eczema, allergic rhinitis, asthma/reactive airway disease, and food allergies. Notably, the allergic triad characterized by increased IgE levels, eosinophilia, and eczema is common among various IEIs, which could delay diagnosis. To date, many complex atopic phenotypes have been described.^10^ Overall, a high grade of severity, even with resistance to standard therapy and/or a combination of different atopic manifestations, has been observed. Moreover, other associated clinical features, “atypical atopic manifestations,” and/or recurrent peculiar, atypical/opportunistic infections should raise the suspicion of a monogenic origin of the atopic disorders.^23^, ^47^, ^48^, ^49^

**Statement #8**. OS is a severe and life‐threatening manifestation of PAD, typical of SCID, that should be quickly ruled out in a newborn or infant with suspected PAD. OS is defined as generalized erythroderma with skin inflammation affecting more than 90% of the body surface, lymphadenopathy, hepatosplenomegaly, profound eosinophilia, chronic persistent diarrhea, failure to thrive, and CID.

*Comment*: Different genetic alterations that significantly reduce without abrogating T cell development and result in an oligoclonal expansion of CD4+ T cells with a consequent exaggerated inflammatory condition typically cause OS. The inflammation is believed to be triggered by clonally expanded T cells, predominantly of the Th2 type, that secrete several cytokines that promote autoimmune and allergic inflammation. Atopic manifestations of OS usually present immediately after birth with generalized erythroderma (skin inflammation affecting more than 90% of the body surface) or eczematous rash. Early recognition is crucial. Some patients present with some but not all of these signs; thus, differential diagnosis can be challenging. Infections, inborn errors of metabolism, ichthyoses, inflammatory skin disorders, and drug hypersensitivity reactions must be ruled out to allow for early HSCT, which is the only curative treatment for this otherwise fatal disease. In OS, skin inflammation progressively worsens, leading to significant barrier damage and increasing the risk of life‐threatening bacterial and fungal infections in an immunocompromised host.^50^

**Statement #9**. The presence of other affected family members, including a family history of IEI and/or familial severe atopic diathesis, and/or a family history of consanguinity could raise the index of suspicion for PAD. Inheritance patterns may differ among distinct IEI with atopic phenotypes.

*Comment*: Accurate collection of familial history may help clinicians to suspect PAD, prioritize relevant investigations, and consider early genetic testing.^5^, ^51^

### 3.5 Laboratory evaluation

**Statement #10**. High levels of Th2 biomarkers and associated immunological abnormalities are of extreme importance in the diagnostic work‐up of IEI with severe atopic features to highlight some indicative features of PAD.

The following exams are mandatory in ruling out PAD in children:

- Complete blood cell count with differential (CBC);
- Serum immunoglobulin (Ig) [IgA, IgM, IgG, IgE], and IgG subclasses dosage;
- Extended T and B cell immunophenotype (CD3, CD4, CD8, CD19, CD56, Treg, Th17, T follicular cells, HLA‐DR expression);
- Specific antibody levels against recall antigens (vaccines, ABO group, others);
- Allergy testing (according to patient's medical history): skin prick test and/or allergen‐specific IgE measurement;
- Markers of organ‐specific and tissue‐specific autoimmunity (according to patient's medical history).

*Comment*: Very few papers suggest which diagnostic tests may be useful to rule out PAD and which are not.^10^, ^11^, ^52^ This statement focuses on first‐ and second‐level investigations in PAD to exploit laboratory signs of immune dysregulation and severe atopy and minimize the probability of underdiagnosis or misdiagnosis. Severe atopy represents the clinical hallmark of PAD, and high Th2 biomarkers and related immune abnormalities are often correlated with severe clinical presentations. Given the overlap of clinical features between PAD and severe atopic diseases, a rational approach is needed to identify other signs of immune dysregulation, which may help differentiate isolated atopy and atopy associated with PAD or other IEI.^53^, ^54^ Combining these tests could allow for a comprehensive immune function evaluation, correlating clinical symptoms (e.g., recurrent infections, severe atopy) with immunological findings for early intervention and management.

**Statement #11**. In the context of severe, especially multiorgan, allergic disease, the following laboratory abnormalities should be considered for the suspicion of PAD:

- cytopenias (neutropenia/thrombocytopenia/anemia);
- lymphopenia;
- increased absolute number of eosinophilic granulocytes (severe if >5000 cells/mm^3^);
- decreased mean platelet volume (MPV);
- very high levels of serum IgE (>1000 kU/L, in the first 3 months of life; >2000 kU/L in children 3–12 months; >5000 kU/L after 12 months of age);
- One or more immunoglobulin isotype deficiency (any combination);
- Low specific antibody levels against protein and polysaccharide antigens (especially after booster vaccination if vaccine antibody response is considered);
- Low Th17 cell counts;
- Low CD3 + CD25+ regulatory T cell counts;
- Very high level of sensitization against multiple environmental allergens (often combined);
- High levels of autoimmunity markers in blood (often precocious and atypical);
- High levels of tryptase in blood.

*Comment*: Integrating clinical presentation—namely severe multiorgan allergic disease—with specific laboratory findings improves early detection of PAD. For example, low Th17 may help differentiate HIES due to *STAT3* dominant‐negative loss‐of‐function mutations from severe atopic dermatitis (AD).^44^, ^55^, ^56^

Cytopenias may indicate bone marrow dysfunction or autoimmune destruction, which can be found in many forms of PAD (e.g., PGM3 deficiency, WAS, WIP deficiency, ARPC1B deficiency, NOCARH, IPEX, *STAT1* GOF). Lymphopenia suggests a T‐cell production or survival defect, often seen in CID (e.g., DOCK8 deficiency, WAS, and WAS‐like syndromes).^57^, ^58^ Eosinophilia, usually associated with exaggerated Th2‐driven organ inflammation, is common in both allergic diseases and many IEIs with atopic phenotypes.^59^ However, hypereosinophilia (i.e., eosinophil counts above 5000 cells/mm^3^) combined with multiorgan dysfunction is uncommon in severe allergic diseases. A low MPV (possibly <6 fL) may suggest megakaryocyte dysfunction, which can be observed in certain PAD (e.g., WAS and WAS‐like conditions).^44^, ^60^ Abnormally elevated IgE levels, associated with somatic signs and/or other symptoms of immune dysfunction, especially in early infancy, indicate specific forms of PAD.^44^, ^61^, ^62^, ^63^, ^64^ Likewise, an inability to produce adequate specific antibodies could orient toward some specific subtypes of PAD.^10^, ^65^, ^66^ In particular, antibody responses should be assessed according to the specific national vaccination schedule and the patient's clinical history. IgG levels against at least three protein antigens (e.g., tetanus and diphtheria toxoids, hepatitis B virus, rubella, mumps, measles) and one polysaccharide antigen (e.g., pneumococcal capsular antigen) should be evaluated. If detected antibody levels are below the normal range, antibody testing should be repeated 4 weeks after administration of a booster dose. Reduced levels of CD3 + CD25+ cells (natural regulatory T cells) suggest impaired immune regulation, often leading to autoimmunity and chronic inflammation. They may suggest IPEX or an IPEX‐like condition in patients with severe atopic dermatitis or other atopic diseases with or without autoimmunity.^67^, ^68^ High sensitization to multiple allergens indicates an overactive Th2‐driven immune response, which can be present in most but not all PAD. However, despite very high IgE levels, some of these conditions may not show any IgE sensitization to environmental allergens or relevant allergic symptoms.^22^, ^23^, ^53^, ^54^, ^69^ High levels of tryptase associated with signs and symptoms of exaggerated and/or abnormal mast‐cell activation may be the prominent feature of some PAD.^5^, ^10^ Finally, early, atypical and/or multisystem/systemic autoimmunity in combination with severe allergic symptoms and elevation of some autoimmunity markers (e.g., organ‐specific auto‐antibodies) is a common feature of many PAD, reflecting immune dysregulation and loss of tolerance.^10^, ^63^, ^70^ These findings suggest immune dysfunction beyond isolated atopy, thus warranting further investigations to rule out PAD. Recognizing these markers ensures timely intervention, avoiding complications and long‐term organ damage.

### 3.6 PAD and skin

**Statement #12**. Atopic dermatitis (AD) represents the typical skin findings of severe atopic disorders as well as of PAD. However, other skin features can be present (ichthyosis, erythroderma, *trichorrhexis invaginata*, ectodermal dysplasia among others).

*Comment*: Focusing on the skin as an affected key organ in PAD requires the consideration that there is a consistent clinical overlap between severe AD and PAD. AD represents a cardinal hallmark of atopy and atopic‐related disorders. It is common in severe atopic disorders, characterized by chronic inflammation, impaired skin barrier function, and a Th2‐skewed immune response. In PAD, AD‐like presentations often co‐occur with recurrent infections, autoimmunity, or other features indicating an underlying immune defect.^71^, ^72^ Additional and broader skin manifestations may suggest specific underlying conditions among PAD. Ichthyosis erythroderma, defined as a generalized scaly and inflamed skin condition, and/or *trichorrhexis invaginata* (hair shaft defect) could be indicative of severe immune dysregulation or syndromic IEIs, such as Comel‐Netherton syndrome.^73^ Furthermore, ectodermal dysplasia, defined as structural abnormalities of the skin and its appendages (hair, nails, teeth), is commonly seen in certain syndromic IEIs, such as HIES or ectodermal dysplasia‐immunodeficiency syndromes [e.g., IKBKG (*NEMO*) deficiency].^74^

**Statement #13**. The following peculiar skin findings may help in differentiating IEI with atopic phenotypes from severe AD:

- Congenital/early onset (even neonatal) erythroderma or neonatal eczematous rash.
- Congenital ichthyosis (early‐onset generalized rash that evolves into severe ichthyosis).
- Bamboo hair (*trichorrhexis invaginata*).
- Atypical localization, extension, and features of lesions (e.g., thickened texture of the facial skin, retro auricular fissures, and severe folliculitis of the axillae and groin).
- Chronic mucocutaneous candidiasis (CMC).
- Cold skin abscesses.
- Warts.
- Ectodermal dysplasia.
- Severe urticarial rash, chronic urticaria, and refractory cold urticaria, with no itching but with pins‐and‐needles‐like pain in affected skin areas
- Striate palmoplantar keratoderma

*Comment*: Medical history and distinctive clinical features are crucial to differentiating severe AD from eczematous lesions caused by IEI. The statement provides a list of skin markers that can help to distinguish PAD from severe AD. The rationale is grounded on clinical clues of immune dysfunction rather than mere atopy.^75^, ^76^ Skin findings such as erythroderma, appearing since birth, the first weeks of life, or early infancy, suggest a SCID or syndromic IEI (e.g., OS and Comel‐Netherton syndrome) rather than typical AD, which usually develops later. Generalized scaling and inflammation that evolve into severe ichthyosis do not suggest AD but rather indicate syndromic IEI like Comel‐Netherton syndrome or disorders with immune dysregulation.^11^, ^77^ Bamboo hair (*trichorrhexis invaginata*) represents a diagnostic marker for Comel‐Netherton syndrome, associated with immune dysregulation and atopic‐like phenotypes.^63^, ^78^ Also, ectodermal dysplasia (structural defects in skin, hair, teeth, and nails) indicates syndromic IEI rather than classic AD. AD typically affects flexural areas (e.g., elbows, knees), but thickened facial skin, retro auricular fissures, and folliculitis in non‐classical areas (axillae, groin) would rather suggest IEI (e.g., HIES).^79^, ^80^ CMC (chronic/persistent fungal infections affecting the skin, mucous membranes, and/or nails) indicates defects in Th17‐mediated immunity, often seen in combined IEI.^56^, ^81^, ^82^, ^83^, ^84^, ^85^ Moreover, recurrent cold skin abscesses (non‐inflammatory, non‐painful) are classic in HIES due to *STAT3* loss of function mutations, while warts and other recurrent or disseminated cutaneous viral infections (e.g. molluscum contagiosum, herpesvirus infections) are common in CID.^73^, ^86^, ^87^, ^88^ In the case of severe urticarial rash and chronic urticaria, the presence of pain instead of itching (pins‐and‐needles sensation) and chronic cold urticaria unresponsive to conventional therapy may point to immune dysregulation, as seen in autoinflammatory syndromes. Finally, striate palmoplantar keratoderma (thickened, striped keratin deposits on palms and soles) is rare in AD but occurs in some syndromic IEI, reflecting skin barrier defects and immune abnormalities.^87^, ^88^, ^89^

### 3.7 PAD and respiratory symptoms

**Statement #14**. PAD should be suspected in severe asthma (also in case of low Th2 endotype), with frequent exacerbations, treatment unresponsiveness, and/or chronic lung disease (including the presence of bronchiectasis). In particular, its occurrence in association with other atypical atopic diseases and/or other features of immune dysregulation should raise the suspicion of PAD and should prompt an immunologist consultation.

*Comment*: Severe and even treatment‐resistant asthma, particularly when in association with features of immune dysregulation, may raise suspicion of PAD. While asthma is a common respiratory condition, its severity, persistence, and treatment resistance may indicate an underlying immune dysfunction. PAD‐related asthma often involves structural lung damage due to recurrent infections or chronic inflammation.^90^, ^91^, ^92^ Severe asthma with a low Th2 inflammatory signature is unusual, but it may suggest immune dysregulation rather than classical allergic asthma, prompting further investigation for PAD. Moreover, recurrent asthma flare‐ups in PAD may result from undiagnosed infections due to impaired immune defense against microbes.^90^, ^91^ Recurrent respiratory infections in PAD often lead to chronic lung damage, including bronchiectasis and pneumatoceles, which are uncommon in typical asthma.^42^, ^90^ Poor response to conventional asthma therapies may suggest an underlying condition. The association of asthma with other atypical atopic features (e.g., severe eczema, severe food allergies) and/or signs of immune dysregulation (e.g., autoimmunity, recurrent infections) strengthens the suspicion of PAD.^33^, ^93^, ^94^ These additional findings highlight a systemic immune dysfunction rather than an isolated respiratory disease. Identifying these signs ensures timely referral, diagnosis, and intervention, reducing morbidity associated with delayed recognition of an underlying IEI.

### 3.8 PAD and food allergy (FA)

**Statement #15**. Multiple and/or severe FA (e.g., anaphylaxis) including food protein‐induced enterocolitis syndrome (FPIES), when associated with other atopic manifestations, especially if severe, and/or other features of immune dysregulation and/or increased susceptibility to infections should raise the suspicion of PAD and should prompt an immunologist consultation.

*Comment*: The statement aims to increase clinicians' surveillance when encountering patients with a combination of severe or multiple FA and signs of immune dysregulation. FA, especially severe ones like anaphylaxis or other conditions like FPIES, suggests an atypical immune response. The presence of multiple FA when they co‐occur with other immune‐mediated manifestations [e.g., increased susceptibility to infections (recurrent or severe infections), other autoimmune or inflammatory conditions] increases the likelihood of an underlying immune disease.^52^, ^93^, ^95^, ^96^, ^97^, ^98^, ^99^ It potentially implies a broader immune dysregulation defect rather than isolated hypersensitivity, pointing toward systemic immune abnormalities rather than isolated allergic conditions.^23^, ^100^, ^101^, ^102^

### 3.9 PAD and eosinophilic gastrointestinal disorders (EGID)

**Statement #16**. EGID associated with severe eosinophilia (>5000 cells/mm^3^) and multiorgan involvement with/without atopic diathesis should be considered as a potential manifestation of PAD. EGID is clinically heterogeneous diseases that are more commonly found in patients with different IEI.

*Comment*: EGID are clinically heterogeneous, presenting with manifestations ranging from mild gastrointestinal symptoms to severe, debilitating disease with systemic involvement. The variability of the clinical presentation underscores the need for thorough evaluation against uncovered underlying conditions, such as some PAD [HIES (*STAT3*‐DN‐HIES, DOCK8 deficiency, Comel‐Netherton Syndrome), ERBIN deficiency, *JAK1* AD GOF, *STAT5b* GOF, and *STAT6* GOF], especially when the disease is severe, atypical, or associated with other atopic conditions (e.g., asthma, eczema, FA).^47^, ^90^, ^93^, ^103^, ^104^, ^105^, ^106^, ^107^, ^108^ Multiorgan involvement in the presence of eosinophilia suggests systemic immune dysregulation rather than an isolated allergic or gastrointestinal issue. The above‐mentioned level of eosinophilia should raise the suspicion of a more profound immune defect.^10^, ^106^

### 3.10 Warning signs

**Statement #17**. Warning signs for PAD include:

- Severe atopic manifestations (especially when multiple) with very early onset (at birth or in the first months of life) and unresponsiveness to standard treatment.
- Recurrent and severe infections due to atypical pathogens (e.g., herpes viruses, HPV, fungi).
- Short stature and/or failure to thrive.
- Lymphoproliferation and/or malignancies.
- Poli‐autoimmunity.
- Severe diarrhea (even with eosinophilic infiltration) and/or enteropathy.
- Poli‐endocrinopathy [with particular regard to early‐onset, eventually neonatal, type 1 Diabetes Mellitus (T1DM)].
- Vascular and connective tissue abnormalities.
- Positive family history for IEI/consanguinity.
- Hematological conditions (e.g., purpura, cytopenia).
- Very high levels of total IgE (>1000 kU/L, in the first 3 months of life; >2000 kU/L in children 3–12 months; >5000 kU/L after 12 months of age).
- Increased absolute number of eosinophils in blood (>5000 cells/mm^3^).

*Comment*: The onset of PAD with severe atopic manifestations may precede the diagnosis by decades.^24^ Timely identification through functional and genetic assays is crucial for optimal management and personalized therapy.^25^, ^41^ Early diagnosis and prompt referral to specialized centers may improve outcomes and can positively influence the life expectancy of patients.^42^ Identifying specific clinical or laboratory findings and developing algorithms that indicate which patients with severe allergies should undergo genetic testing for IEI is crucial.^25^ Early‐onset, atypical, and/or recalcitrant atopic disease, often in conjunction with immunodeficiency and/or autoimmunity, should raise the suspicion of an IEI.^109^ Recurrent and severe infections, particularly with unusual pathogens (e.g., fungi, herpes viruses, HPV), suggest compromised immune function.^86^, ^110^, ^111^, ^112^ Short stature and/or failure to thrive reflect chronic illness, malabsorption (e.g., due to enteropathy), or persistent inflammation stemming from an immune system abnormality.^110^, ^112^, ^113^, ^114^ Chronic immune activation in severe atopic conditions can predispose to abnormal lymphocyte proliferation or malignancies due to prolonged inflammation and immune dysregulation.^110^, ^115^, ^116^ Autoimmune phenomena can coexist with severe atopic disorders, highlighting a broader dysfunction of immune regulation that affects both hypersensitivity and tolerance mechanisms. Chronic or severe gastrointestinal symptoms may result from immune‐mediated inflammation, including eosinophilic infiltration, or infections due to weakened mucosal immunity.^13^, ^117^, ^118^ Early‐onset autoimmune diabetes and other endocrine dysfunctions (poli‐endocrinopathy) may indicate systemic autoimmunity, commonly seen in IEI.^67^, ^119^, ^120^, ^121^ Certain IEIs, such as HIES, present with connective tissue or vascular abnormalities and/or dysmorphic features.^114^, ^122^, ^123^ A family history of inherited immune dysregulation or severe atopic disorders suggests a genetic predisposition to IEI.^124^ Familial cases or consanguinity increase the likelihood of inherited immune deficiencies.

Cytopenia or abnormal bleeding patterns often indicate autoimmune or hematological conditions associated with an IEI.^119^ High levels of total IgE and eosinophilia, particularly in young children, are hallmarks of certain PAD, such as HIES syndromes and immune signaling defects.^9^, ^125^, ^126^, ^127^

**Statement #18**. The combination of severe atopic manifestations (especially when multiple) with two or more of the following clinical and laboratory findings increases the possibility of a PAD:

- Very early onset (<3 months).
- Recurrent and severe infections due to atypical pathogens (e.g., herpes viruses, HPV, fungi).
- Short stature and/or failure to thrive.
- Lymphoproliferation and/or malignancies.
- Poli‐autoimmunity.
- Severe diarrhea (even with eosinophilic infiltration) and/or enteropathy.
- Poli‐endocrinopathy (with particular regard to early‐onset, eventually neonatal T1DM).
- Vascular and connective tissue abnormalities.
- Positive family history for IEI/consanguinity.
- Very high levels of total IgE (>1000 kU/L, in the first 3 months of life; >2000 in children 3–12 months; >5000 kUL after 12 months of age).
- Increased absolute number of eosinophils in blood (>5000 cells/mm^3^).
- Cytopenia along one or more lineages.

*Comment*: The combination of severe atopic manifestations and the listed clinical and laboratory findings may help recognize immune dysregulation and systemic features characteristic of PAD. PAD involves severe atopic conditions that often coexist with broader immune dysfunctions, including autoimmunity, infections, and hematological or systemic abnormalities.^5^, ^8^, ^9^, ^10^, ^25^, ^41^, ^95^, ^96^, ^115^, ^116^, ^120^, ^121^, ^128^ The combination of these features increases the likelihood of a PAD diagnosis, reflecting systemic immune dysregulation beyond isolated atopic diseases. The combination of severe atopy with these systemic and laboratory findings demonstrates a continuum of immune dysregulation beyond typical allergic conditions. Recognizing this combination allows for the early diagnosis of PAD, enabling timely interventions to prevent complications.

**Statement #19**. The combination of severe atopic manifestations (especially when multiple), including urticaria, angioedema, and anaphylaxis, with one or more of the following laboratory findings, once a malignant hematological disorder has been excluded, increases the possibility of a PAD:

- Very high levels of total IgE (any age) >10,000 kU/L.
- Increased absolute number of eosinophils in blood (any age) >10,000 cells/mm^3^.
- Basal high levels of tryptase and/or histamine in blood.

However, lower values of these biomarkers may be commonly found in IEI associated with atopy and do not rule out a PAD.

*Comment*: Combining the above‐listed clinical features and specific laboratory markers may help identify PAD. Markedly elevated total IgE and severe eosinophilia are uncommon in isolated allergic conditions and may indicate a more significant immune dysregulation. Elevated basal tryptase or histamine levels may reflect mast cell hyperactivation and dysregulation, occurring in some PAD.^5^, ^9^, ^19^, ^89^, ^90^ However, conditions like eosinophilic leukemia can also present with similar laboratory findings. Hence, it is critical to rule these out before attributing the findings to PAD. Lower biomarker levels than the proposed cut‐off do not rule out PAD but might require additional context or diagnostic testing to confirm the diagnosis.

**Statement #20**. Normal levels of total IgE do not rule out a diagnosis of PAD.

*Comment*: PAD is a heterogeneous condition with many different immune pathways involved. Cases of PAD with severe hypereosinophilia without significant elevation of IgE have been reported.^108^, ^129^ Although the serum IgE levels are frequently elevated, there are cases of PAD (even among HIES) with only mildly elevated IgE or within the normal range, depending on the underlying genetic defect and phenotype. Total IgE level is influenced by multiple factors, including genetics, environmental factors, and the presence of coexisting conditions.^61^, ^65^, ^98^ Thus, it is not universally elevated in all cases of PAD. While elevated total IgE can be a clue in the diagnosis of some PAD, it is not a definitive or necessary marker. Clinicians must consider normal IgE levels in the context of other clinical and laboratory findings, avoiding over‐reliance on this single parameter in diagnosing PAD.^108^, ^129^

### 3.11 Genetics

**Statement #21**. Identification of warning signs for PAD should prompt an immunologic consultation prior to genetic testing. Moreover, the indication and type of genetic testing should be given as a joint geneticist and clinical immunologist evaluation [expert opinion].

*Comment*: Even in the next‐generation and whole exome sequencing era, clinical suspicion and immunologist consultation should drive the subsequent diagnostic work‐up, usually requiring functional testing and genetic analysis. This may be crucial in this expanding group of IEI, where a few cases of some newly discovered diseases have been described, and the same genes may be involved both in common type 2 disease and in true immunodeficiencies with an atopic phenotype.^12^ Therefore, an immunologist should always give a final diagnosis with the joint consultation of a clinical geneticist.

**Statement #22**. Clinical response to specific target disease modifying therapies is expected once genetic diagnosis has been established and pathogenetic mechanisms elucidated [expert opinion].

*Comment*: Monogenic atopic disorders represent an emblematic example of precision medicine, in which the knowledge of pathogenic mechanisms offers several targeted therapeutic options. On the other hand, good clinical response to a specific targeted therapy supports the involvement of the mechanism, but it does not differentiate between the monogenic rather than the multifactorial nature of the disease, as it may also be obtained in non‐monogenic atopic disorders.^32^, ^34^, ^36^, ^107^, ^117^, ^118^, ^130^, ^131^, ^132^, ^133^, ^134^, ^135^, ^136^, ^137^, ^138^, ^139^, ^140^, ^141^, ^142^, ^143^

**Statement #23**. As for other IEI, novel disease‐causing genes are continuously identified for PAD. For these reasons, an initial negative result does not rule out PAD diagnosis, and patients should be periodically re‐evaluated according to the most recent knowledge.

*Comment*: In cases where a strong suspicion of PAD exists, re‐evaluation by an immunologist and geneticist might pave the way to a definite diagnosis as the clinical phenotype may vary over time. Many reports exist of patients with a long delay before diagnosis.^8^, ^39^, ^51^, ^55^, ^93^, ^144^, ^145^, ^146^, ^147^, ^148^, ^149^, ^150^

**Statement #24**. Functional tests should be used to confirm PAD diagnosis and evaluate the pathogenic role of variants of uncertain significance (VUS) eventually detected. Functional tests could also drive a therapeutic approach while waiting for the genetic analysis results.

*Comment*: Genetic analyses may identify VUS in genes involved in innate and adaptive immune responses linked to atopic pathways, but their significance is not always straightforward. Functional tests bridge the gap between clinical data and genetic results. They are essential to confirm PAD diagnosis, understand the clinical impact of VUS, and guide treatment decisions while awaiting genetic results. This integrated approach ensures accurate diagnosis and timely intervention.^55^, ^93^, ^145^, ^151^, ^152^

## 4 DISCUSSION

Lyons and Milner first coined the definition of PAD in their seminal paper published in 2018 to collect a heterogeneous group of inborn immune disorders with a broad clinical spectrum characterized by extremely skewed T2 immune response and/or abnormal mast‐cell activation.^5^ Most affected individuals present with long‐lasting disabling diseases, starting early in life, requiring multiple hospitalizations or outpatient evaluations by different specialty clinics before diagnosis. Quality of life is often poor because of difficult‐to‐treat symptoms with multiorgan involvement, and growth and development are often compromised. However, in the era of precision medicine, new targeted therapies have dramatically changed the clinical course of patients affected by some forms of PAD. Therefore, increased awareness and timely diagnosis of this expanding group of newly discovered diseases is desirable. So far, many excellent reviews focusing on PAD have been published by different groups of researchers, but diverse approaches by different specialists presumably still exist when facing the same disease.^9^, ^11^, ^12^, ^25^, ^26^, ^41^ We attempted to standardize the approach to such a multifaceted group of disorders by different experts involved in managing organ‐specific symptoms typical of PAD. This was done by prioritizing the identification of potential red flags and the development of a shared management, specifically focusing on the diagnostic work‐up.

In the present consensus, a Delphi approach was applied to discuss a core set of 24 statements to systematize clinicians' approach to these disorders. An optimal level of agreement >80% after the first round was obtained for all but one (statement 22) statement. However, given the complexity of the topic and the need to reach a degree of agreement as great as possible among different specialists to fully harmonize the management of PAD, a higher‐than‐usual level of agreement was sought and set at >85%. Further, comments by panelists about two statements (statements 5, 15), even when passing the arbitrary threshold of >85% of agreement, were judged as “relevant” by the advisory board and allowed to be voted on the second round. Overall, five statements (statements 5, 8, 15, 19, 22) were voted on again after panelist comments, reaching the requested threshold of agreement. Unexpectedly, after the second round, a decrease in the level of agreement was registered in one statement (statement 5, passing from 97.62% to 90.5% level of agreement). However, the chosen threshold of agreement was reached, whilst the other (statement 15) remained substantially unchanged. The former result could probably be explained by the difficulty some immunologists have in accepting the collection of such a kaleidoscope of IEI, overlapping with many other immune dysregulation disorders, under a unique “umbrella” entity. An example of this heterogeneity is represented by the inclusion of mast cell activation disorders among PAD. In this context, systemic mastocytosis is most often a clonal disorder driven by somatic activating mutations in the *KIT* gene. However, its genetic background is complex, and many experts consider that somatic *KIT* gain‐of‐function mutations alone do not fully account for the variability in the clinical phenotype of the disease. Indeed, additional germline variants in *KIT* and in genes encoding for cytokines or their receptors (e.g., IL13, IL6, IL6R, IL31, IL4R), as well as increased copy numbers of the *TPSAB1* gene encoding for α‐tryptase, have been increasingly recognized as associated with systemic mastocytosis in adults.^153^

This consensus presents some limitations. One limitation is the overrepresentation of immunologists among the panelists. All the components of the Italian Pediatric Expert Task Force on Primary Atopic Disorders (F.C., I.T., M.S., L.P., V.M., C.C., R.B., R.C.) were members of the SIAIP, thus reflecting mainly an allergists' and immunologists' perspective on PAD. However, in our country, most patients affected with PAD are managed primarily in pediatric immunology or allergy outpatient clinics. Moreover, geneticists and qualified members of other Italian pediatric societies were included among the panelists, and they fruitfully contributed to the discussion of each statement. Given the tremendous heterogeneity in the phenotype of PAD, the recent discovery of some diseases, and the difficulty in extrapolating a set of clinical and laboratory findings sensitive and specific enough to establish a core of “red flags” (“one‐fits‐all”), another limitation is that some statements were based on simple expert opinions. Finally, the proposed shared management is limited to the diagnostic work‐up and only one statement (statement 22) refers to the therapeutic approach. This reflects the challenges of defining a standardized treatment strategy for conditions characterized by marked clinical and molecular heterogeneity. As for the therapeutic options available for PAD, we have recently provided a comprehensive summary of the current literature in Taietti et al.^154^

**FIGURE 3:**
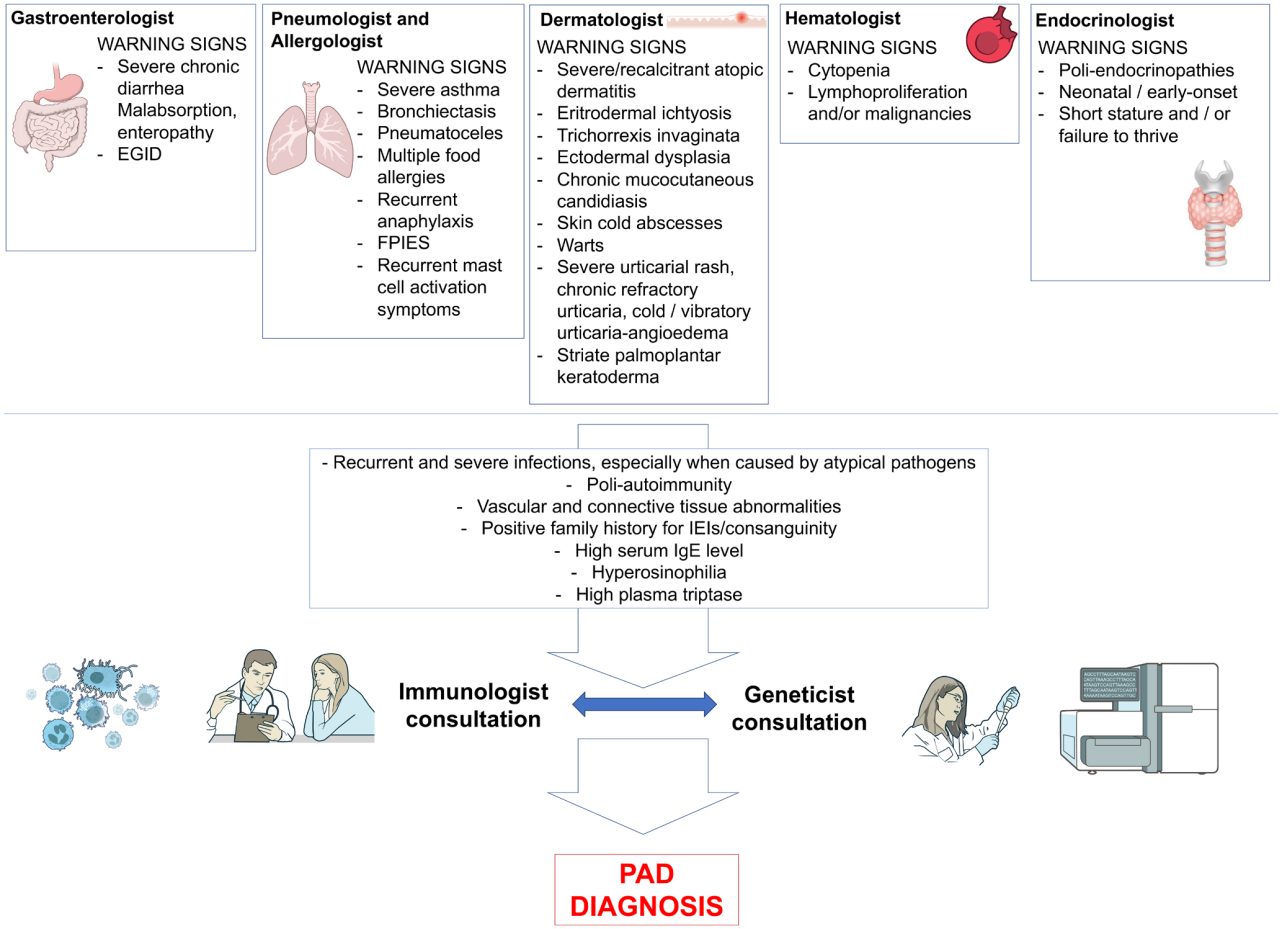
Proposal for a multidisciplinary approach for suspected Primary Atopic Disorders. EGID, eosinophilic gastrointestinal disease; FPIES, food protein‐induced enterocolitis syndrome.

On the other hand, the major strength of the document was that, as far as we know, it is the first published document that aims to establish a shared perspective among pediatric subspecialists in caring for patients affected by PAD. Figure 3 shows a proposal for a multidisciplinary pediatric approach.

In conclusion, PAD represent a group of IEI being continuously updated, requiring a standardized approach from pediatricians and all specialists involved in the care of these conditions. Appropriate PAD knowledge among clinicians, including multifaceted clinical symptoms and the proper work‐up and therapeutic approach, will pave the way for a timely diagnosis before deteriorating organ dysfunction is established.

## AUTHOR CONTRIBUTIONS

**Fabio Cardinale:** Conceptualization; investigation; validation; writing – review and editing; project administration; visualization; supervision; writing – original draft. **Ivan Taietti:** Writing – original draft; methodology; investigation; writing – review and editing; data curation. **Mayla Sgrulletti:** Writing – original draft; writing – review and editing. **Lucia Pacillo:** Writing – original draft; writing – review and editing. **Viviana Moschese:** Writing – review and editing; supervision. **Caterina Cancrini:** Writing – review and editing; supervision. **Raffaele Badolato:** Writing – review and editing; supervision. **Michele Miraglia del Giudice:** Project administration; supervision. **Gian Luigi Marseglia:** Project administration; supervision. **Elena Chiappini:** Methodology; writing – review and editing; supervision. **Riccardo Castagnoli:** Writing – original draft; writing – review and editing; investigation; data curation; supervision; conceptualization; validation; project administration.

## FUNDING INFORMATION

Nothing to declare.

## CONFLICT OF INTEREST STATEMENT

The authors declare no conflict of interest.

## PEER REVIEW

The peer review history for this article is available at https://www.webofscience.com/api/gateway/wos/peer‐review/10.1111/pai.70225.

## Appendix 1 Primary Atopic Disorders Working Group

### 1.1

Primary Atopic Disorders Working Group: Salvatore Barberi (Pediatric Unit, Rho and Garbagnate Milanese Hospital, ASST‐Rhodense, Milan, Italy), Anna Belloni Fortina (Regional Center of Pediatric Dermatology and Genodermatoses, Department of Woman's and Child's Health SDB, University of Padua, Italy), Roberto Berni Canani (Department of Translational Medical Science, University of Naples “Federico II”, Naples, Italy), Paolo Bottau (Pediatric and Neonatology Unit, Imola Hospital, Imola, Italy), Carlo Caffarelli (Department of Medicine and Surgery, Clinica Pediatrica, Azienda Ospedaliero Universitaria, University of Parma, Parma, Italy), Mauro Calvani (Operative Unit of Pediatrics, S. Camillo‐Forlanini Hospital, Rome, Italy), Antonella Cianferoni (Allergy and Immunology Division, Perelman School of Medicine, The Children's Hospital of Philadelphia, University of Pennsylvania, Philadelphia, Pennsylvania, USA), Francesco Cinetto (Rare Diseases Referral Center, Internal Medicine 1, Department of Medicine (DIMED), AULSS2 Marca Trevigiana, Ca' Foncello Hospital, University of Padova, Padova, Italy), Bianca Laura Cinicola (Department of Maternal, Infantile and Urological Sciences, Sapienza University of Rome, Rome, Italy), Emilia Cirillo (Department of Translational Medical Sciences, Pediatric Section, Federico II University, Naples, Italy), Francesca Conti (Pediatric Unit, IRCCS Azienda Ospedaliero‐Universitaria di Bologna, Bologna, Italy; Department of Medical and Surgical Sciences, Alma Mater Studiorum, University of Bologna, Bologna, Italy), Laura Dotta (Pediatrics Clinic and Institute for Molecular Medicine A. Nocivelli, Department of Clinical and Experimental Sciences, University of Brescia and ASST‐Spedali Civili of Brescia, Brescia, Italy), Marzia Duse (Department of Maternal, Infantile and Urological Sciences, Sapienza University of Rome, Rome, Italy), Silvia Federici (Division of Rheumatology, Bambino Gesù Children's Hospital, IRCCS, Rome, Italy), Alessandro Fiocchi (Allergy Unit, Bambino Gesù Children's Hospital, IRCCS, Rome, Italy), Elena Galli (UOS Immuno–Allergologia Pediatrica, Ospedale S. Pietro Fatebenefratelli, Rome, Italy), Silvia Giliani (‘A. Nocivelli’ Institute for Molecular Medicine, Department of Molecular and Translational Medicine, University of Brescia, Brescia, Italy), Giuliana Giardino (Department of Translational Medical Sciences, Pediatric Section, Federico II University, Naples, Italy), Cristiana Indolfi (Department of Woman, Child and of General and Specialized Surgery, University of Campania “Luigi Vanvitelli”, Naples, Italy), Lucia Leonardi (Department of Maternal, Infantile and Urological Sciences, Sapienza University of Rome, Rome, Italy), Amelia Licari (Pediatric Unit, Department of Clinical, Surgical, Diagnostic, and Pediatric Sciences, University of Pavia; Pediatric Clinic, Fondazione IRCCS Policlinico San Matteo, Pavia, Italy), Vassilios Lougaris (Pediatrics Clinic and Institute for Molecular Medicine A. Nocivelli, Department of Clinical and Experimental Sciences, University of Brescia and ASST‐Spedali Civili of Brescia, Brescia, Italy), Sara Manti (Pediatric Unit, Department of Human Pathology in Adult and Developmental Age “Gaetano Barresi”, University of Messina, Messina, Italy), Alberto Martelli (Italian Society of Pediatric Allergy and Immunology (SIAIP), Italy), Domenico Minasi (Pediatric Unit of Ospedali Riuniti Presidium, Grande Ospedale Metropolitano Bianchi Melacrino Morelli, Reggio Calabria, Italy), Elio Novembre (Department of Health Sciences, University of Florence, Florence, Italy), Riccardo Papa (UOC Rheumatology and Autoinflammatory Diseases, IRCCS Istituto Giannina Gaslini, Genova, Italy), Claudio Pignata (Department of Translational Medical Sciences, Pediatric Section, Federico II University, Naples, Italy), Federica Pulvirenti (Centre for Primary Immune Deficiencies, Azienda Ospedaliera Universitaria Policlinico Umberto I, Rome, Italy), Maria Sangerardi (Department of Pediatric Emergency Medicine, Policlinico – “Giovanni XXIII” Pediatric Hospital of Bari, University of Bari, Italy), Annarosa Soresina (Pediatrics Clinic and Institute for Molecular Medicine A. Nocivelli, Department of Clinical and Experimental Sciences, University of Brescia and ASST‐Spedali Civili of Brescia, Brescia, Italy), Mariangela Tosca (Allergy Unit IRCCS Istituto G. Gaslini Genoa, Italy), Alberto Giovanni Ugazio (Institute of Child and Adolescent Health, Bambino Gesù Children's Hospital, IRCCS, Rome, Italy), Anna Maria Zicari (Department of Maternal, Infantile and Urological Sciences, Sapienza University of Rome, Rome, Italy).
